# Vertebral artery trauma in a stab wound to the ear: case report^☆^

**DOI:** 10.1016/j.bjorl.2016.05.007

**Published:** 2016-06-20

**Authors:** Sha Jichao, Meng Cuida, Zhu Dongdong

**Affiliations:** China-Japan Union Hospital of Jilin University, Department of Otorhinolaryngology Head and Neck Surgery, Changchun, China

## Introduction

Traumatic vertebral artery injury to the ear is uncommon. Although the majority of patients with vertebral artery injuries are hemodynamically stable, serious hemorrhage and death are possible. In this paper, a case is reported of trauma to the vertebral artery caused by a stab wound to the ear. Endovascular intervention was performed after surgical exploration and angiographic examination. We learned that angiography may be essential in some situations for patients with penetrating trauma to the ear, especially when active or recurrent hemorrhage continues after surgical exploration.

## Case report

A 36 year-old man presented to our Otorhinolaryngological Department, 5 days after surgical exploration of a stab wound to the left ear, with a recurrent hemorrhage that centered on the intertragic notch.

Debridement, external carotid artery ligation, internal jugular vein ligation, and superficial parotidectomy were performed immediately after his presentation in a local hospital. At admission, his blood pressure was 105/74 mm/Hg and heart rate 78 beats per minute, and he had no neurological deficits. Physical observations found a swollen face and active bleeding from an S-shaped incision, which was about 10 cm, centered on the intertragic notch. Further examinations showed that the tympanic membrane and eyes were normal, while the ipsilateral marginal mandibular branch of the facial nerve was injured.

A prophylactic tracheotomy was performed to prevent possible tracheal compression due to the large hematoma. However, we did not find the precise bleeding location, after deep re-exploration in the cervical vertebral area. Therefore, injury to the vertebral artery was suspected.

**Figure 1:**
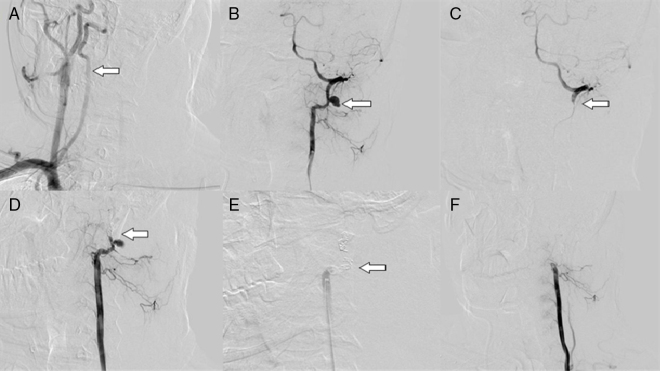
The angiographic examination and endovascular therapy for the case of traumatic vertebral artery in a stab wound to the ear. (A) The angiogram showed that the right vertebral artery was normal. (B) The injury and bleeding was located. (C) Distal embolization of the affected vertebral artery. (D) Interpretation of the distal blood flow. (E) Proximal embolization of the affected vertebral artery. (F) Confirmation of total embolization.

An angiography revealed partial laceration of the left vertebral artery of the segment located between C1 and C2. Subsequently, primary endovascular intervention was performed (Fig. 1). The procedure proved to be successful and no active bleeding or symptoms to the cerebral ischemia were detected.

The patient was discharged 12 days after the procedure. The patient signed the consent forms and agreed to dedicate the findings to this case report. The local ethics committee approved the protocol of this case study.

## Discussion

There are many reports of successful treatment of traumatic vertebral artery performed through endovascular intervention.^1^, ^2^ However, clinical descriptions regarding an injury to the vertebral artery caused by trauma to the neck are uncommon, particularly for injury to the ear. Studies have shown that 74% of patients with vertebral injuries caused by a penetrating neck wound presented only stable hematoma and no other clinical findings.^3^ In the present case report, recurrent and active hemorrhage occurred despite surgical intervention conducted in a local hospital. Indeed, in cases of trauma to the vertebral artery, the complexity of anatomical features, distortion arising from trauma, and hemorrhage may complicate surgical intervention.

The patient in this case received ligature of the external carotid artery and jugular vein during primary surgical exploration, and a surgical re-exploration from the staff in our Otorhinolaryngological Department. Unfortunately, the source of the bleeding could not be located. Injury to the vertebral artery was therefore highly suspected and angiography was performed. Angiography is considered effective for identifying arterial injuries, and for simultaneous endovascular interventions for vascular lesions, which are difficult to find through regular surgical exploration.^4^ Moreover, cerebral angiography is indispensable because it allows identification of variant anatomy; and the exclusion of additional vessel injuries, including to the contralateral vertebral artery or ipsilateral carotid artery, and distal vertebral artery occlusion. Cerebral angiography also allows evaluation of the compensatory ability of the contralateral artery, by positioning of the transarterial balloon catheter, before transient embolization of the injured vertebral artery. In addition cerebral angiography also makes placement of embolism materials easier, as near as possible to the injury, both proximal and distal.^5^

For the patient described in this report, it was speculated that the path of injury was the knife's tip piercing from the intertragic notch to the mandibular trailing edge. First, the knife tip went into the pterygomandibular space, then pierced the lateral stylohyoid muscle, continued to the transverse process of the cervical vertebra, and finally penetrated into the vertebral artery. No associated cervical spine injury was detected. It was confirmed angiographically that the contralateral vertebral artery did not have any malformation and was capable of providing sufficient blood supply to the brain; then an embolization was performed.

According to similar case reports, the majority of patients do not develop any related ischemic damage to neural tissue after vertebral artery ligation.^6^ However, a few patients have exhibited signs of nervous system infarction, and even death after unilateral vertebral artery ligation.^7^ The patient in the present case report had no recurrent hemorrhage or symptoms of cerebral blood supply insufficiency after endovascular intervention. This indicated that the procedure was effective, and unilateral vertebral artery was not associated with brain ischemia. This may be because, within the 5 days of recurrent ischemia prior to angiography, the wound to the vertebral artery did not hinder blood flow.

In the present case, the bleeding was slower than would be expected from an external carotid artery, and first surgical intervention was ineffective. This suggests a pseudoaneurysm of the vertebral artery, secondary to a laceration of the vessel adventitia.

Considering these characteristics, we should be alert to the possibility of vertebral artery injury when treating patients with a stab wound to the ear, particularly for patients with recurrent bleeding after surgical exploration. In these clinical situations, angiographic examination and subsequent endovascular therapy should be considered earlier.

To the best of our knowledge, the literature is very limited regarding injury to the vertebral artery that is associated with ear trauma. Our experience with the present case may alert otolaryngologists to possible vertebral artery injury in ear trauma. When a partial laceration is clearly identified, endovascular intervention is the preferred treatment and ultimately may lead to reduced risk of mortality.

## Conclusion

We recommend that possible injury of the vertebral artery should be suspected, and angiography may be necessary, for patients with penetrating trauma to the ear.

## Conflicts of interest

The authors declare no conflicts of interest.

## References

[1] Mei Q., Sui M., Xiao W., Sun Z., Bai R., Huang C. (2014). Individualized endovascular treatment of high-grade traumatic vertebral artery injury. Acta Neurochir (Wien).

[2] Pejkic S., Ilic N., Dragas M., Dimic A., Koncar I., Cvetkovic S. (2014). Indirect surgical management of a penetrating vertebral artery injury. Vascular.

[3] Reid J.D., Weigelt J.A. (1988). Forty-three cases of vertebral artery trauma. J Trauma.

[4] Nicolau C., Gilabert R., Chamorro A., Vazquez F., Bargallo N., Bru C. (2000). Doppler sonography of the intertransverse segment of the vertebral artery. J Ultrasound Med.

[5] Yee L.F., Olcott E.W., Knudson M.M., Lim R.C. (1995). Extraluminal, transluminal, and observational treatment for vertebral artery injuries. J Trauma.

[6] Hoshino Y., Kurokawa T., Nakamura K., Seichi A., Mamada T., Saita K. (1996). A report on the safety of unilateral vertebral artery ligation during cervical spine surgery. Spine (Phila Pa 1976).

[7] Burke J.P., Gerszten P.C., Welch W.C. (2005). Iatrogenic vertebral artery injury during anterior cervical spine surgery. Spine J.

